# String Grammar Unsupervised Possibilistic Fuzzy C-Medians for Gait Pattern Classification in Patients with Neurodegenerative Diseases

**DOI:** 10.1155/2018/1869565

**Published:** 2018-06-13

**Authors:** Atcharin Klomsae, Sansanee Auephanwiriyakul, Nipon Theera-Umpon

**Affiliations:** ^1^Computer Engineering Department, Faculty of Engineering, Chiang Mai University, Chiang Mai, Thailand; ^2^Biomedical Engineering Institute, Chiang Mai University, Chiang Mai, Thailand; ^3^Excellence Center in Infrastructure Technology and Transportation Engineering, Chiang Mai University, Chiang Mai, Thailand; ^4^Electrical Engineering Department, Faculty of Engineering, Chiang Mai University, Chiang Mai, Thailand

## Abstract

Neurodegenerative diseases that affect serious gait abnormalities include Parkinson's disease (PD), amyotrophic lateral sclerosis (ALS), and Huntington disease (HD). These diseases lead to gait rhythm distortion that can be determined by stride time interval of footfall contact times. In this paper, we present a new method for gait classification of neurodegenerative diseases. In particular, we utilize a symbolic aggregate approximation algorithm to convert left-foot stride-stride interval into a sequence of symbols using a symbolic aggregate approximation. We then find string prototypes of each class using the newly proposed string grammar unsupervised possibilistic fuzzy C-medians. Then in the testing process the fuzzy k-nearest neighbor is used. We implement the system on three 2-class problems, i.e., the classification of ALS against healthy patients, that of HD against healthy patients , and that of PD against healthy patients. The system is also implemented on one 4-class problem (the classification of ALS, HD, PD, and healthy patients altogether) called NDDs versus healthy. We found that our system yields a very good detection result. The average correct classification for ALS versus healthy is 96.88%, and that for HD versus healthy is 97.22%, whereas that for PD versus healthy is 96.43%. When the system is implemented on 4-class problem, the average accuracy is approximately 98.44%. It can provide prototypes of gait signals that are more understandable to human.

## 1. Introduction

Neurodegenerative diseases (NDDs) are the diseases of neuronal destruction in the central nervous system. The NDDs cause the volume of the brain and the amount of nerve deterioration over time. The diseases reduce the ability of patient and destroy tissue and nerves of the brain because nerves or neurons in the brain normally cannot reproduce themselves. Some neurodegenerative disorders such as Parkinson's disease (PD), Huntington disease (HD), and amyotrophic lateral sclerosis (ALS) usually occur at an older age and can lead to serious gait abnormalities [1]. Since balancing and sequencing of movement are controlled by the central nervous system, the gait of patient with neurodegenerative disorders will become abnormal. The main symptoms of PD are legs trembling, slowed moving, and impaired posture and balance. It may grow worse over time [2]. The main symptoms of HD are mood change, coordination of muscles problem, uncontrolled movement, and difficulty in walking. The patient with HD may lose their intellectual and behavioural abilities and may also experience psychiatric symptoms [3]. For ALS patient, a part of nerve cells that control muscle function is destroyed. Characteristic of this disease is continuous muscle atrophy. It causes muscle weakness and tenderness. The general symptoms in ALS are difficulty in walking, swallowing, breathing, and speaking [4]. In [5], they found that the patients with neurodegenerative diseases had decreased stride length as compared to healthy control subjects. From above reasons, the stride-to-stride of gait information is utilized for gait pattern classification in patients with neurodegenerative diseases because of the gait pattern difference between healthy and NDD subjects.

In recent related studies, the information from time series of stride intervals, swing intervals, and stance intervals of stride-to-stride is utilized to classify the gait pattern of the patient with NDDs and healthy control subjects. Some research works involved detecting either PD or ALS only [9, 13, 14]. Some of them involved HD, ALS, and PD classification [8, 10–12]; however, the information from left and right feet is used in the system. A few of them utilized only right-foot information to classify HD, ALS, and PD [7]; however, this method only detected a patient with one disease against a healthy patient, not finding a patient with one of the diseases against a healthy patient. All previous researches utilized a regular numeric classifier, e.g., the support vector machine and K*∗* classifier. Hence, these methods cannot provide a prototype signal for each disease.

**Figure 1:**
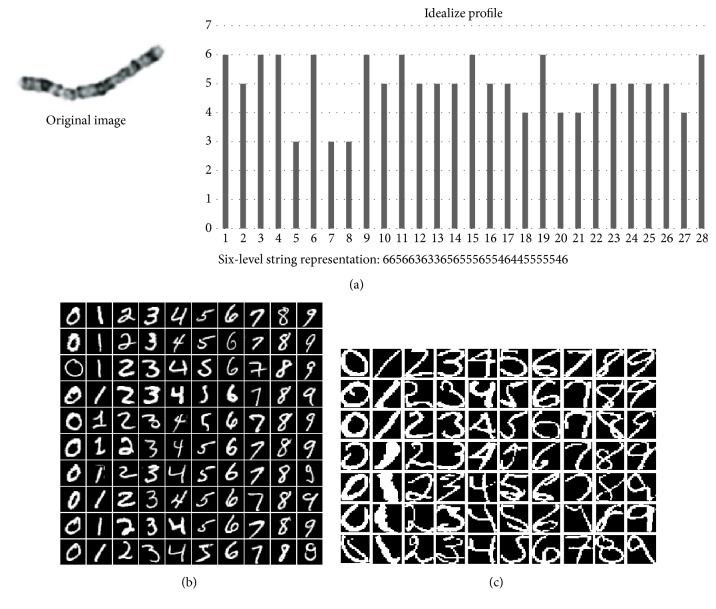
An example from (a) the Copenhagen chromosomes data set, (b) the MNIST data set, and (c) the USPS data set.

In this paper, we propose the syntactic method for gait pattern classification from time series information. In particular, we introduce a string grammar unsupervised possibilistic fuzzy C-medians (sgUPFCMed) to recognize PD, ALS, and HD from the left-foot stride interval. It is worthwhile noting that the sgUPFCMed is a brand new algorithm proposed by our research group. It is a part of the recent doctoral thesis of one of our group members [6] and has never been published elsewhere. In the thesis, it was implemented on some standard data sets that are syntactic data set by nature, e.g., the Copenhagen chromosomes data set [15–17], the MNIST database of handwriting digit data set from http://algoval.essex.ac.uk/data/sequence/ as described in [18–21] collected by Professor Simon M. Lucas, and the USPS handwritten digit data set collected by Professor Simon M. Lucas and downloaded from http://algoval.essex.ac.uk/data/sequence/ [18–21]. Example from each data set is shown in Figure 1. The histogram of each image in the Copenhagen chromosomes data set was encoded into a string. It should be noted that we downloaded the encoded data set, not the images in these three data sets. The experiment results on both 10-fold cross validation and the blind test data sets from all three data sets are shown in Table 1. This shows that the algorithm is capable of classifying syntactic data set and also providing good classification results.

Since our algorithm is not a numeric classifier but a syntactic classifier, we transform the gait time series into a string using the symbolic aggregate approximation (SAX) [22]. The sgUPFCMed is utilized to find a string prototype(s) for each disease. Then the fuzzy k-nearest neighbor [23] is utilized to find the best match for a test data sample. The paper is structured as follows. The description of the NDDs detection system is introduced in Section 2. The results of gait classification are shown in Section 3. Finally, we draw the conclusion in Section 4.

## 2. System Description

**Figure 2:**
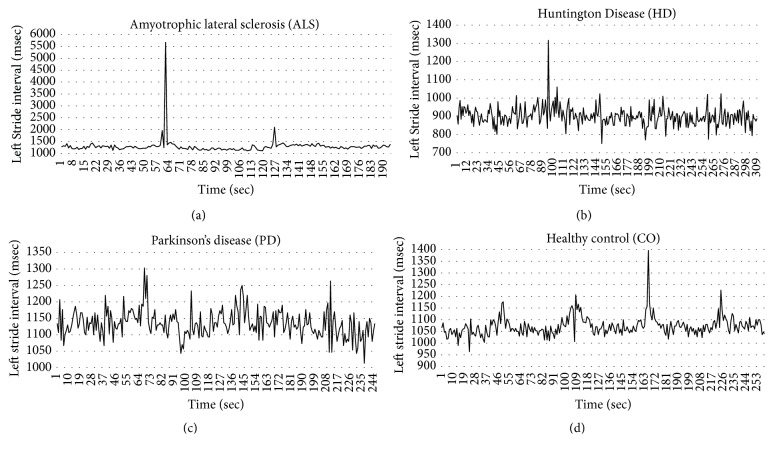
An example of sequence of stride times from different groups of subjects including (a) a subject with ALS disease, (b) a subject with HD, (c) a subject with PD, and (d) a healthy control (CO) subject.

**Figure 3:**
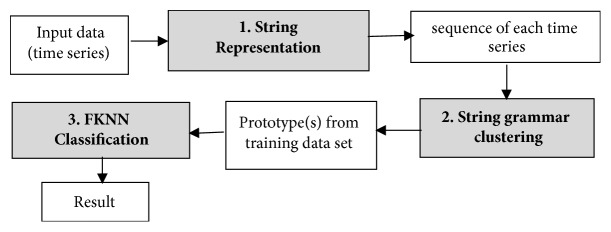
System overview of gait patterns classification in patients with neurodegenerative diseases.

In this section, we introduce the details of our system for gait pattern classification of patients with neurodegenerative diseases (NDDs). We take the gait data set from gait dynamics in neurodegenerative disease database (http://www.physionet.org/physiobank/database/gaitndd/). This data set consists of 64 subjects from 15 subjects with PD, 20 subjects with HD, 13 subjects with ALS, and 16 healthy control subjects [24]. Subjects were requested to walk along a 77-meter-long hallway for 5 minutes without stopping. Force-sensitive switches underneath each subject's feet were recorded at 300 Hertz sampling rate. From the recorded force, the time series of the stride time, stance time, and swing time were derived. To eliminate the startup effects, we follow the same method in [25]. The first 20 values of each samples are removed. The 3-SD median filter is utilized for eliminating the outliers that are far away from the median value [25]. The raw data are obtained using force-sensitive resistors, with the output roughly proportional to the force under the foot. Stride-to-stride measures of footfall contact times are derived from these signals as shown in Figure 2. In the experiment, we only use left-foot stride-to-stride interval data set. The proposed scheme of the detection system is shown in Figure 3. We transform each time series data into a sequence string using the symbolic aggregate approximation (SAX) representation [22] to convert any time series into a sequence of symbols. The gait time series $\overset{⃑}{T}$ of length* n* is converted into its Piecewise Aggregation Approximation (PAA) (a vector of* w*-dimensional space (${\overset{⃑}{P}}_{i}=p_{1},\ldots ,p_{w}$)) using

(1) $$\begin{array}{c} {\overset{⃑}{P}}_{i}=\frac{w}{n}\overset{\left(n/w\right)i}{\underset{j=\left(n/w\right)\left(i-1\right)+1}{\sum }}p_{j}. \end{array}$$

**Figure 4:**
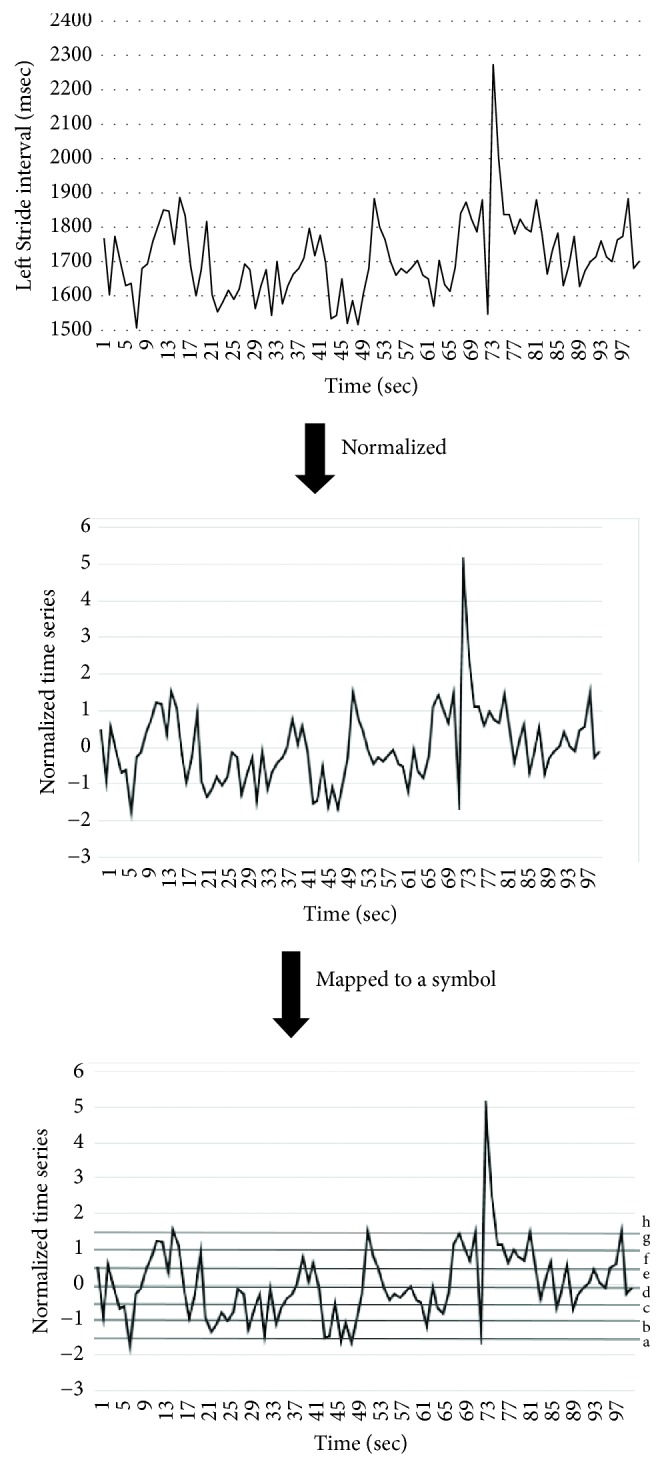
Example of string generation from gait time series.

The time series data ($\overset{⃑}{T}$) is normalized into a series data with 0 mean and 1 standard deviation. Then it is divided into several frames with the size of* w* and each frame is converted to PAA data (${\overset{⃑}{P}}_{i}$). Then each ${\overset{⃑}{P}}_{i}$ (for *i* = 1,…, ⌊*n*/*w*⌋) is mapped into a symbol. In our experiment,* w* is set to be equal to the length of the time series. There are 8 symbols used in the experiment. Example of the string generation is shown in Figure 4. In this figure the gait time series is transformed to “fbfdbcaddfgh……dffhdd”.

Now, we are ready to create prototypes with the string grammar unsupervised possibilistic fuzzy clustering (sgUPFCMed). The sgUPFCMed is a modified version of the unsupervised possibilistic fuzzy C-means (UPFCM) [26], a combination of the possibilistic fuzzy C-means (PFCM) [27] and the unsupervised possibilistic clustering (UPCM) [28]. It is to solve the problem of generating coincident clusters of the UPCM. The UPFCM is developed based on the characteristics of both fuzzy and possibilistic C-means. Hence, the UPFCM should be able to deal more effectively with noise, overlapping, and outliers. Since the sgUPFCMed is modified from the UPFCM, it should have the same properties as the UPFCM. The brief description of the algorithm is as follows. Assume *S* = {*s*_1_, *s*_2_,…, *s*_*N*_} be a set of *N* strings. Each string (*s*_*k*_) is a sequence of symbols (primitives). For example, *s*_*k*_ = (*x*_1_*x*_2_ … *x*_*l*_), a string with length *l*, where each *x*_*i*_ is a member of a set of defined symbols or primitives. Suppose **V** = (*sc*_1_, *sc*_2_,…, *sc*_*C*_) represents a *C*-tuple of string prototypes, each of which characterizes one of the *C* clusters. *Lev*(*sc*_*i*_, *s*_*j*_)] is the Levenshtein distance [29–32] between string *s*_*j*_ and string prototypes *sc*_*i*_.** U** is a membership matrix [*u*_*ik*_]_*C*×*N*_ and** T** is a possibilistic matrix [*t*_*ik*_]_*C*×*N*_. The objective function of the sgUPFCMed is

(2) min⁡Jm,ηU,T,V;S=∑i=1C ∑k=1Nauikm+btikηLevsk,sci+βη2c∑i=1C ∑k=1Ntikηlog⁡tikη−tikη,

where *u*_*ik*_ is the membership value of string *s*_*k*_ in the cluster *i*, *t*_*ik*_ is the possibilistic value of string *s*_*k*_ in the cluster *i*, *m* is the fuzzifier (normally *m* > 1), *η* > 1, *β* > 0, *a* > 0, *b* > 0, ∑_*c*=1_^*C*^*u*_*ik*_ = 1 for *k* = 1,…, *N*, and 0 ≤ *u*_*ik*_, *t*_*ik*_ ≤ 1. *β* is defined as the sample covariance [23] based on the Euclidean distance. Since our data set is a string data set, the calculation of *β* will be

(3) $$\begin{array}{c} \beta =\frac{\sum_{k=1}^{N}Lev\left(Med,s_{k}\right)}{N}, \end{array}$$

where* Med* is the median string of the data set; i.e.,

(4) $$\begin{array}{c} Med=\underset{j\in S}{arg\;min}\overset{N}{\underset{k=1}{\sum }}Lev\left(\;s_{j},s_{k}\;\right)\;\text{for }1\leq i\leq C. \end{array}$$

The theorem for the sgUPFCMed and its corresponding proof are shown in Theorem 1. This theorem shows that the update equation of a membership value of string *k* in cluster *i* (*u*_*ik*_) (5) and the update equation of a possibilistic value of string *k* in cluster *i* (*t*_*ik*_) (6) give the minimum value of the objective function (*J*_*m*,*η*_(**U**, **T**, **V**; *S*)).

**Theorem 1 (sgUPFCMed).**

If *Lev*(*s*_*k*_, *sc*_*i*_) > 0 for all *i* and *k*, when *m*, *η*, *k* > 1, and *S* contains *C* < *N* distinct string data, then *J*_*m*,*η*_ is minimized only if the update equation of *u*_*ik*_ is

(5) $$\begin{array}{c} u_{ik}=\frac{1}{\sum_{j=1}^{C}{\left(Lev\left(sc_{i},s_{k}\right)/Lev\left(sc_{j},s_{k}\right)\right)}^{1/\left(m-1\right)}} \end{array}$$

and the update equation of *t*_*ik*_ is

(6) $$\begin{array}{c} t_{ik}=\exp\left(\;-\frac{b\eta \sqrt{c}Lev\left(sc_{i},s_{k}\right)}{\beta }\;\right). \end{array}$$

**Proof**

From the Lagrange multiplier theorem, (5) is obtained by solving the reduced problem min_**U**∈*M*_*fcn*__⁡{*J*_*m*,*η*_^*k*^(**U**) = ∑_*i*=1_^*C*^(*au*_*ik*_^*m*^ + *bt*_*ik*_^*η*^)*Lev*(*s*_*k*_, *sc*_*i*_)} where** T** and** V **are fixed for the* k*-th column of** U**. The proof of this equation is similar to that in [23]; hence, it is obvious and easy to prove (5).

Similarly, when** U **and** V** are fixed for the* i*-th row of** T**, (6) is proved by solving the problem $\min\left\{L_{i}\left(T,\lambda \right)=J_{m,\eta }^{ik}(T)=\left(au_{ik}^{m}+bt_{ik}^{\eta }\right)Lev\left(s_{k},sc_{i}\right)+(\beta /\eta^{2}\sqrt{c})\sum_{i=1}^{C}\sum_{k=1}^{N}\left(t_{ik}^{\eta }\log t_{ik}^{\eta }-t_{ik}^{\eta }\right)\right\}$. The derivative of *L*_*i*_(**T**, *λ*) with respect to *t*_*ik*_ and setting it to zero leads to

(7) ∂LiT,λ∂tik=bηtikη−1Levsk,sci+βη2cη2tikη−1ln⁡tik=0

(8) bηcLevsk,sci+βln⁡tikc=0

(9) tik=exp⁡−bηcLevsci,skβ.

To update a cluster center, we utilized the fuzzy median string [23, 33–36] as follows:

(10) sci=arg minj∈S⁡∑k=1Nauikm+btikηLevsj,skfor  1≤i≤C.

However, it has been proved in [35, 36] that the modified median string provides a better classification than the regular median string. Hence, in [23, 33–36], the modified fuzzy median string is used. Let Σ*∗* be the free monoid over the alphabet set Σ and a set of strings *S*⊆Σ*∗*. Then, the modified fuzzy median, i.e., an approximation of fuzzy median using edition operations (insertion, deletion, and substitution) over each symbol of the string, will be

(11) sci=arg minj∈Σ∗⁡∑k=1Nauikm+btikηLevsj,skfor  1≤i≤C.

The cluster center update equation of the sgUPFCMed is shown in Algorithm 1.

The sgUPFCMed algorithm is summarized in Algorithm 2.

Afterwards, the multiprototype generation, i.e., *SC* = {*sc*_1_^1^,…, *sc*_*N*_1__^1^, *sc*_1_^2^,…, *sc*_*N*_2__^2^, *sc*_1_^*C*^,…, *sc*_*N*_*C*__^*C*^}, where *sc*_*k*_^*j*^ is string prototype *k* of class *j*, is created. The fuzzy k-nearest neighbor (FKNN) [23, 37] is used as a classifier. The membership value *u*_*i*_ of string *s* in class *i* is

(12) $$\begin{array}{c} u_{i}\left(\;s\;\right)=\frac{\sum_{j=1}^{K}u_{ij}{\left(1/Lev\left(sc_{j}^{q},s\right)\right)}^{1/\left(m-1\right)}}{\sum_{j=1}^{K}{\left(1/Lev\left(sc_{j}^{q},s\right)\right)}^{1/\left(m-1\right)}} \end{array}$$

where *u*_*ij*_ is the membership value of the *j*^th^ prototype from class *q*(*sc*_*j*_^*q*^) in class *i*, *c* is the number of classes, and *K* is the number of nearest neighbors. The decision rule for the test string *s* is

(13) $$\begin{array}{c} s\text{ is assigned to class }i\;\text{if }u_{i}\left(s\right)>u_{j}\left(s\right)\text{ for }j\neq i. \end{array}$$

Because the class of each prototype is known, we set membership value to 1 for *sc*_*j*_^*q*^ in class *q* and zero membership values in all other classes.

## 3. Experiment Results

**Figure 5:**
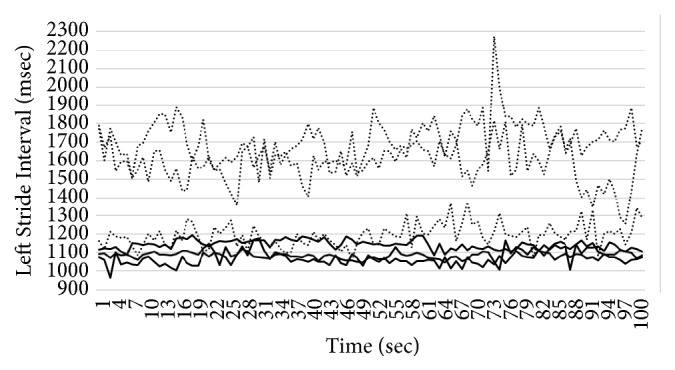
Closest time series to the prototypes of ALS and healthy patient.

**Figure 6:**
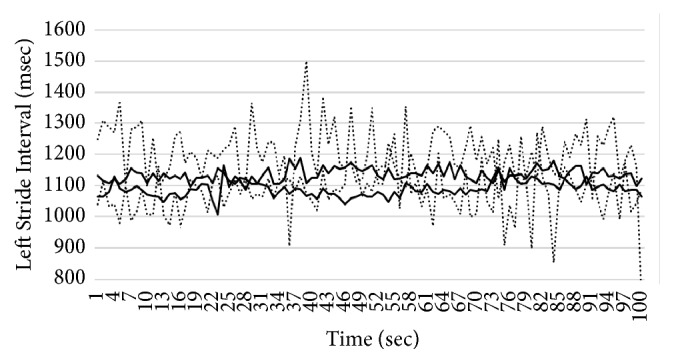
Closest time series to the prototypes of HD and healthy patient.

**Figure 7:**
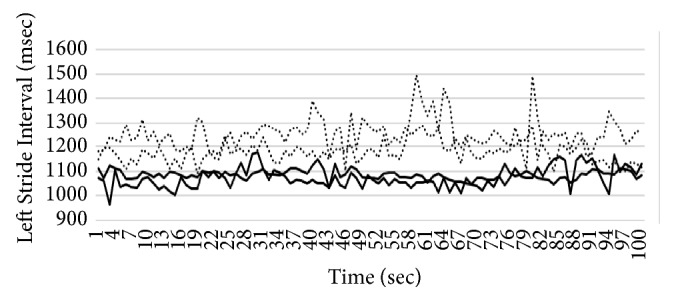
Closest time series to the prototypes of PD and healthy patient.

**Figure 8:**
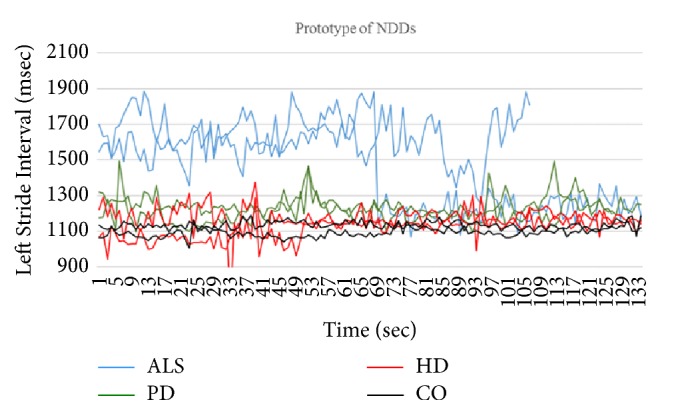
Closest time series to the prototypes of NDDs and healthy patient.

We implement three 2-class problems, i.e., the classification of ALS against healthy patients, HD against healthy patients, and PD against healthy patients. We also implement one 4-class classification, i.e., the classification of all three NDDs diseases (ALS, HD, and PD) against healthy patients. In all of the experiments, we implement 4-fold cross validation to evaluate our proposed algorithm. The parameters *m* and *η* are set to 2, and the parameters *a* and *b* are set to 1 and 6, respectively. These parameters are chosen based on trial and error method from an extensive experiment. The stopping criteria of the sgUPFCMed are set to 0.01 with the maximum number of iterations of 100. To create multiprototype of each class, the sgUPFCMed is used to cluster each class with 2, 3, 4, and 5 number of clusters. In the testing process, the FKNN is utilized with *K* = 1, 3, and 5. Tables 2–5 show the average and the standard deviation of the classification rate on the validation set for the ALS versus healthy, HD versus healthy, PD versus healthy, and NDDs versus healthy. The best validation result from the ALS is 96.875±6.250% when there are 3 prototypes for each class and 1 nearest neighbor, while that from the HD is 97.222±5.556% with 2 prototypes for each class and 1 nearest neighbor. The best result from the PD is 96.429±7.143% with 2 prototypes and 1 nearest neighbor. For all three NDDs classes versus healthy patient, the best result is again 2 prototypes and 1 nearest neighbor with the classification rate of 98.437±3.125%. The sensitivity and specificity of the best model in ALS, HD, PD, and NDDs are shown in Table 6. Figures 5–8 show time series that are closest to prototypes of the best model of the ALS, HD, PD, and NDDs classification experiment, respectively. We can see that the shape of each prototype is not exactly similar to the others. Although, there are some overlapping between prototypes of the disease gait signal and the healthy gait signal, the detection system can provide a good classification rate. For example, in Figure 6, the prototypes of HD gait signals are overlapped with that of the healthy control prototypes.

However, the shapes are different. The string sequences will be different as well. Hence, the classification result is close to 100%. We also compare our results indirectly with the existing methods as shown in Table 7. We can see that our results are better than the numeric algorithms in all the cases except PD and HD classification in 2-class problem and NDDs in 4-class problem. However, the algorithm in [8] was implemented using all-train-all-test whereas our result is based on the validation set only. The algorithm in [12] used several features while our system only uses left-foot stride-to-stride interval. Moreover, our system can provide the shapes of prototypes that might be more understandable to user than the numeric algorithms.

## 4. Conclusions

In this paper, the NDDs, i.e., Parkinson's disease (PD), amyotrophic lateral sclerosis (ALS), and Huntington Disease (HD), detection system is introduced. In particular, the NDDs left-foot gait time series (left-foot stride-stride interval) is transformed into a sequence of strings. The string grammar unsupervised possibilistic fuzzy C-medians (sgUPFCMed) first introduced in this paper is utilized to generate prototypes of each disease. Then the fuzzy k-nearest neighbor is used as a classifier in the testing process. We found that the best validation results of the 2-class problem, i.e., ALS versus healthy patient, HD versus healthy, and PD versus healthy, are 96.88±6.25%, 97.22±5.56%, and 96.43±7.14%, respectively. For the 4-class problem (three NDDs versus healthy), the best classification rate is 98.44±3.13%. From the indirect comparison, we found that our algorithm performs better than the existing algorithms on average. In addition, our system can provide the prototype signal that is more understandable to human than the previous methods that are based on numeric algorithm.

## Figures and Tables

**Algorithm 1:**
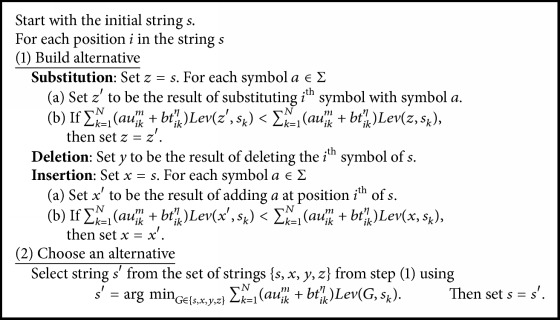


**Algorithm 2:**
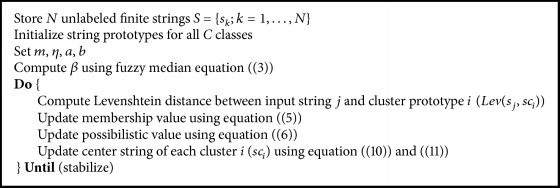


**Table 1:** Results from 3 data sets [6].

Data set	Validation sets	Blind test data set
	Average error ± standard deviation (%)	Average error ± standard deviation (%)
Copenhagen chromosomes	87.05%±1.23%	87.82%±1.65%
MNIST	97.89%±0.21%	98.09%±0.44%
USPS	95.53%±0.31%	93.46%±0.91%

**Table 2:** The average ± standard deviation of classification rate of ALS versus healthy validation set.

# prototypes (*p*) of each class	*k* of FKNN		
	1	3	5
2	93.304±7.767	79.018±8.794	-
3	**96.875±6.250**	89.732±6.897	69.643±14.725
4	92.857±8.248	92.857±8.248	86.161±11.698
5	89.732±6.897	93.304±7.767	79.018±8.794

**Table 3:** The average ± standard deviation of classification rate of HD versus healthy validation set.

# prototypes (*p*) of each class	*k* of FKNN		
	1	3	5
2	**97.222±5.556**	88.889±12.830	-
3	91.667±10.638	88.889±9.072	77.778±20.286
4	91.667±10.638	83.333±11.111	83.333±14.344
5	80.556±5.556	83.333±6.415	80.556±16.667

**Table 4:** The average ± standard deviation of classification rate of PD versus healthy validation set.

# prototypes (*p*) of each class	*k* of FKNN		
	1	3	5
2	**96.429±7.143**	74.553±13.937	-
3	90.179±12.156	83.929±15.636	77.679±11.527
4	90.179±12.156	87.054±17.700	77.679±11.527
5	87.054±10.245	87.054±10.245	80.804±12.231

**Table 5:** The average ± standard deviation of classification rate of NDDs versus healthy validation set.

# prototypes (*p*) of each class	*k* of FKNN		
	1	3	5
2	**98.437±3.125**	90.625±8.069	-
3	92.188**±**5.984	90.625±6.250	89.063±9.375
4	90.625**±**3.608	87.50±7.217	85.938±9.375
5	85.938±9.375	84.375±8.069	82.820±9.375

**Table 6:** Sensitivity and specificity of ALS, HD, PD, and NDDs detection.

	Sensitivity	Specificity
ALS versus healthy	100.00±0.00	93.75±12.50
HD versus Healthy	95.00±10.00	100.00±0.00
PD versus Healthy	93.75±12.50	100.00±0.00
NDDs versus healthy	97.92±4.17	93.75±12.50

**Table 7:** Comparison of the proposed method with the existing methods.

Method	Classification error rate (%)
ALS versus Healthy (2-class problem)	
Our proposed method	**96.88±6.25**
Symbolic entropy [7]	82
Radial basis function (RBF) neural networks (All-training-all-testing) [8]	93.1
Radial basis function (RBF) neural networks (Leave-one-out) [8]	89.66
Least squares support vector machine (Leave-one-out) [9]	82.8
Radial basis function (RBF) support vector machines [10]	96.79
Meta-classifier [11]	96.1326

HD versus Healthy (2-class problem)	
Our proposed method	**97.22±5.56**
Symbolic entropy [7]	95
Radial basis function (RBF) neural networks (All-training-all-testing) [8]	100
Radial basis function (RBF) neural networks (Leave-one-out) [8]	83.33
Radial basis function (RBF) support vector machines [10]	90.23
Meta-classifier [11]	88.674

PD versus Healthy (2-class problem)	
Our proposed method	**96.43±7.14**
Symbolic entropy [7]	89
Radial basis function (RBF) neural networks (All-training-all-testing) [8]	100
Radial basis function (RBF) neural networks (Leave-one-out) [8]	87.1
Radial basis function (RBF) support vector machines [10]	89.33
Meta-classifier [11]	90.3581

NDDs versus Healthy (4-class problem)	
Our proposed method	**98.44±3.13**
Radial basis function (RBF) neural networks [8]	93.75
K*∗* classifier [12]	99.17
DECORATE [12]	94.69
Random Forest [12]	94.69
Radial basis function (RBF) support vector machines [10]	90.63

## Data Availability

The data set is downloaded from http://www.physionet.org/physiobank/database/gaitndd/. It is a public data set provided by physionet.org.
